# Mobilizing the research ecosystem for scientific advances towards positive impact in the context of the COVID-19 Pandemic

**DOI:** 10.3906/sag-2004-180

**Published:** 2020-04-21

**Authors:** Hasan MANDAL

**Affiliations:** 1 The Scientific and Technological Research Council of Turkey, Ankara Turkey

**Keywords:** COVID-19, SARS-CoV-2

## Abstract

This special issue of the *Turkish Journal of Medical Sciences* is dedicated to providing scientific advances in the process of better understanding the SARS-CoV-2 virus that causes the COVID-19 infection. The special issue is published in a special time in which science-based approaches, cocreation-based collaboration, and the effective utilization and integration of competences have a crucial role during the race against time while combating the COVID-19 pandemic. In this process, the Scientific and Technological Research Council of Turkey (TÜBİTAK), which publishes academic journals including the *Turkish Journal of Medical Sciences*, has taken rapid action to mobilize the research community. This includes forming new scientific coalitions in record time, the opening of new calls across the research ecosystem, the organization of a virtual scientific conference, and the launch of a new portal in support of cocreation processes and open science. In addition, various teleconferences that bring together various disciplines at the national and international level have taken place. All of these efforts provide multiple venues to support the common effort of combating the COVID-19 pandemic with R&D and development as a common objective. The sharing of evidence-based knowledge and scientific progress is an effective approach towards providing important contributions for combating the COVID-19 pandemic. The research articles that are contained in this special issue of the *Turkish Journal of Medical Sciences* involves a special collection dedicated to COVID-19. This short communication aims to provide an introduction of the major initiatives that have been taken in the scientific landscape with a focus on Turkey.

**Cocreation in the COVID-19 Turkey Platform for Vaccine and Drug Development**

The importance that TÜBİTAK has given to the establishment of cocreation models has been represented in such initiatives as the TÜBITAK 1004 Call for High Technology Platforms prior to the COVID-19 pandemic. The first phase of 17 High Technology Platforms that were already launched in the leadership of research universities and research centers with competence under Law 6550 on Supporting Research Infrastructure had also included platforms on drug and vaccine development. In particular, diagnostic kits, drug and vaccine development including those for influenza-based infections as well as bioindicator and high technology drug products and prototype vaccines had taken place among the focus areas of the research programs of these platforms.^1^1The Scientific and Technological Research Council of Turkey (2018). Research Programs with Support Under the High Technology Platforms Call Phase 1 [online]. Website https://tubitak.gov.tr/sites/default/files/1776/1004_arastirma_programlari_listesi_0.pdf [accessed 14 April 2020] (in Turkish).

As a fast track option with a particular focus on COVID-19, a subplatform under the coordination of TÜBİTAK Marmara Research Center (MAM) Genetic Engineering and Biotechnology Institute has been established, namely the COVID-19 Turkey Platform. The subplatform brings together projects and competencies that can be transformed for an effective response against the COVID-19 infection with a particular focus on medicine, vaccines, and innovative treatment approaches. Remarkably, the orchestration of researchers for the COVID-19 Turkey Platform and the time for the implementation of the projects have been completed in a record time of only 10 days.^2^2COVID-19 Turkey Web Portal (2020). COVID-19 Turkey Platform Vaccine and Drug Development Virtual Conference is Organized [online]. Website https://covid19.tubitak.gov.tr/duyurular/covid-19-turkiye-platformu-asi-ve-ilac-gelistirme-sanal-konferansi-duzenledi [accessed 15 April 2020] (in Turkish). 

Such a scientific coalition brings together researchers from universities, public R&D units, and the industry together to work on drug repurposing, drug development, innovative treatment approaches, and vaccine development against COVID-19. The platform currently involves 7 different vaccine and 8 different drug development projects where both chemical and biotechnological methods are applied. Multiple methods that have the potential of enabling an effective response in support of combating the COVID-19 infection are undertaken. For the grand goal of supporting an effective, science-based response to the COVID-19 pandemic, hundreds of researchers from 24 universities, 8 public R&D units, and 8 private sector firms are working diligently. There are currently 225 researchers with tasks in the platform of which 116 are from universities, 62 are from public R&D units, and 47 are from the private sector. Overall, the platform involves a total of 15 projects that are being supported for a duration of up to 9–12 months.

The Health Institutes of Turkey (TÜSEB) under the Ministry of Health is collaborating to support the clinical trials of platform developments. This collaboration will enable the projects that are supported under the COVID-19 Turkey Platform to be translated into new products and treatments. 

Recent updates from the COVID-19 Turkey Platform can be summarized based on the following:

- In silico modeling studies of FDA approved or Turkey Pharmaceuticals and Medical Devices Agency (TITCK) licensed drugs are undertaken to determine drug repurposing candidates that are to be tested for COVID-19 treatments. These studies are initiated to test the effectiveness of such drugs and to test their effectiveness based on in vitro studies.

- Virus isolation for SARS-CoV-2 is accomplished in Turkey, first by the Head of the Department of Virology of Ankara University Faculty of Veterinary Medicine and Director of the Institute of Biotechnology who is responsible for the project “Development of Griffithin-Based Antiviral Approach for the COVID-19 and Investigation of its Effectiveness” under the COVID-19 Turkey Platform, with additional virus isolation results taking place.

- Important advances have been made for the application of convalescent plasma for critically ill COVID-19 patients. Studies at Acıbadem Labcell and İstanbul Medeniyet University have reached the final stage. Detection of donors and appropriate plasma donations are being checked. Transfusion to the patients will begin following the arrival of ELISA kits for the determination of plasma RT-QPCR and COVID-19 IgG/IgM titers.

- Together with İstanbul Medeniyet University, viral RNA is obtained from samples taken from patients and sequencing studies are initiated by TÜBİTAK MAM Genetic Engineering and Biotechnology Institute (GMBE). In the study, the SARS-CoV-2 virus genome that has been isolated by Ankara University is in the process of being sequenced. 

- qPCR tests designed by TÜBİTAK MAM GMBE have been completed and validated by comparison with commercial kits. The institute is able to perform COVID-19 qPCR tests or screening when needed and this capability has been shared with the Ministry of Health.

- Neutralizing antibody library scans were initiated by obtaining RNA in the biosafety level BSL3 lab from blood from patients and healthy individuals in the development project of neutralizing antibodies against COVID-19. Collection of COVID-19 samples are ongoing.

**COVID-19 Turkey Platform Vaccine and Medicine Development Virtual Meeting **

On April 2, 2020, The Ministry of Industry and Technology of the Republic of Turkey and TÜBİTAK organized “The COVID-19 Turkey Platform Vaccine and Medicine Development Virtual Meeting” that focused on drug repurposing, drug development, innovative treatment approaches, and vaccine development. In total, 14 speakers attended the meeting that was arranged in 3 sessions. The meeting was livestreamed online via YouTube, Twitter, and Facebook for the access of the public. The livestreaming received a very high interest with about 130,000 viewers. 

The virtual conference included opening statements from the Minister of Industry and Technology and the President of TÜBİTAK. The opening remarks underlined the importance of science-based solutions based on cocreating together and achieving together within the research ecosystem. In particular, the first session of the virtual conference was held with a focus on “Drug-Repurposing and Drug Development,” which was followed by the second session on “Convalescent Plasma and New Generation Treatment Methods.” The third session included presentations on “Vaccines ”.^3^3COVID-19 Turkey Web Portal (2020). COVID-19 Turkey Platform Vaccine and Drug Development Virtual Conference [online]. Website https://covid19.tubitak.gov.tr/duyurular/covid-19-turkiye-platformu-asi-ve-ilac-gelistirme-sanal-konferansi [accessed 15 April 2020] (in Turkish).

**A New Virtual Portal – The COVID-19 Turkey Web Portal**

TÜBİTAK recognizes the importance of utilizing virtual environments effectively in the process of combating COVID-19. For this reason, a first-of-its-kind web portal was established that includes scientific developments regarding COVID-19 as well as a clear and transparent reporting of daily statistics (Figure). The virtual scientific portal is established by the Ministry of Industry and Technology of the Republic of Turkey and TÜBİTAK, namely the COVID-19 Turkey Web Portal.^4^4Ministry of Science and Technology of the Republic of Turkey and TÜBİTAK (2020), COVID-19 Turkey Web Portal [online]. Website https://covid19.tubitak.gov.tr/ [accessed 15 April 2020] (in Turkish).  The portal includes recent developments, the competences of the Turkish ecosystem in related disciplines and sectors, and a Scientific Sharing Platform for competent researchers. Open science resources with a particular focus on COVID-19, including open-access publications, open access collections, and open data sets, are included from both national and international databases. Related research calls of TÜBİTAK, TÜSEB, the European Commission, and others are also announced. The portal has received more than 1.6 million visits from 210 thousand visitors since March 26, 2020.

**Figure 1:**
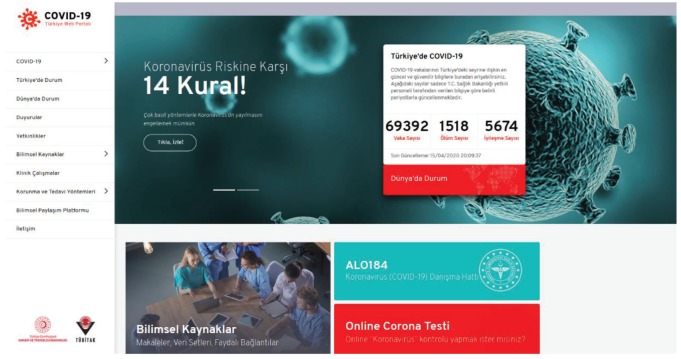
COVID-19 Turkey Web Portal 4.

**Intern Researcher Scholarship Program (STAR) for COVID-19 Research Projects**

Another important aspect of mobilizing the research ecosystem for supporting a science-based combat with the COVID-19 pandemic is through motivating human resources. A rapid call is launched with a focus on supporting the participation of young scholars in COVID-19 related research topics while reaching important milestones of the research projects. Undergraduate, graduate, doctorate, and postdoctoral researchers are provided the opportunity of being supported with the Intern Researcher Scholarship Program (STAR) to participate in projects in the fight against COVID-19. The call aims to contribute to the fight against COVID-19, to minimize and control the spread of the epidemic in society, to protect public health, especially risk groups, and to mitigate the negative socio-economic effects of the epidemic.^5^5The Scientific and Technological Research Council of Turkey (2018). Intern Researcher Scholarship Program (STAR) [online]. Website https://www.tubitak.gov.tr/tr/duyuru/covid-19-ile-mucadeleye-genclerimizi-de-dahil-ediyoruz [accessed 15 April 2020] (in Turkish). From the perspective that the “coronavirus is not stronger than the measures that are taken,” the call aims to increase the potential of young researchers to cooperate in the research ecosystem. The COVID-19 Projects Scholar Demand Interface was also created on the COVID-19 Turkey Web Portal to provide an opportunity for candidates who wish to apply under the call to meet with demands in COVID-19 projects.^6^6COVID-19 Turkey Web Portal (2020). COVID-19 Projects Scholar Demand Interface [online]. Website https://covid19.tubitak.gov.tr/covid-19-projeleri-bursiyer-talepleri-arayuzu [accessed 15 April 2020] (in Turkish).

**Increased Interaction between Researchers Across Disciplines and Countries**

A teleconference was held between representatives of the COVID-19 Turkey Platform and members of the Coronavirus Science Committee of the Ministry of Health of the Republic of Turkey, including experts in rheumatology and hematology, with the aim of sharing experiences and discussing common needs. At the international level, a teleconference was held with representatives of Academia Sinica as the science academy of Taiwan. TÜBİTAK is dedicated to increasing interactions between researchers across disciplines and countries during COVID-19. 

**The Rapid COVID-19 Call under the Support Program for SMEs**

TÜBİTAK established a rapid funding mechanism for SMEs that have research activities on COVID-19 diagnosis, protective and medical equipment as well as ICT solutions.^7^7The Scientific and Technological Research Council of Turkey (2018). Call for Combating COVID-19 [online]. Website https://www.tubitak.gov.tr/tr/duyuru/1507-covid-19 [accessed 15 April 2020] (in Turkish). As another rapid response measure, the special, fast track call was effective for a duration of 1 week for the project applications while the next 1 week duration for the project evaluation continues. The entire process from call launch to the determination of the supported projects will last 2 weeks. The call focused on COVID-19 to support projects for the development of products that can be used in the diagnosis and treatment of COVID-19 and protective equipment that are effective in the prevention of the disease. The applications for the call were much higher than expected. In total, 444 project applications for the call were received from SMES in the scope of combating COVID-19.^8^8The Scientific and Technological Research Council of Turkey (2018). Great Interest for the Call on Combating COVID-19 [online]. Website https://www.tubitak.gov.tr/tr/duyuru/covid19-cagrisina-buyuk-ilgi [accessed 15 April 2020] (in Turkish). Beyond the target of funding 10 projects, a total of 35 projects received support decisions after evaluation.

The call covered the following solutions but did not exclude other COVID-19 related proposals. The funded projects will receive support lasting up to 9 months, enabling a fast track to market.

· Disinfectants

· Masks

· Protective clothing

· Diagnostic kits with verification criteria (sensitivity, specificity, accuracy, precision, linearity) within internationally accepted limits

· Devices used in intensive care units, such as ventilators 

· Equipment for improving ambient conditions 

· Drugs 

· Vaccines

· IT applications that may reduce the direct or indirect consequences of the epidemic

**Mobilization of Social Sciences Research on the Impacts of the COVID-19 Pandemic**

In addition to medicinal sciences, TÜBİTAK will launch a call to reveal the impacts of the COVID-19 pandemic on socio-economic, social, and industrial developments. In the scope of social effects, the focus will include behavioral changes, equality of opportunity, accessibility of work, education, food supply, communication as well as psychological and sociological changes. R&D projects will cover studies that focus on determining the current status as well as short-, medium-, and long-term projections, forecasting and analysis studies as well as foresight studies. These inputs are targeted to be used in creating solutions to be taken by public institutions, the private sector and research institutions regarding economic and social welfare in the future.

**Summary of the Science-Based Response to the COVID-19 Pandemic**

The science-based response to the COVID-19 pandemic that has been implemented by TÜBİTAK involves multiple fronts. Throughout the process, a key emphasis has been placed in the realization of the cocreation process that is supported by evidence-based and qualified knowledge production as well as qualified human resources. Open science approaches have been utilized in this process to support the knowledge base for the cocreation process, including a new virtual portal, namely the COVID-19 Turkey Web Portal. The web portal has also been effective in sharing recent scientific developments as well as the knowledge sources from the virtual conference. TÜBİTAK is glad to publish a special issue of the *Turkish Journal of Medical Sciences* that is dedicated to the sharing of scientific advances in the special time of a COVID-19 pandemic.

